# Identification and genetic characteristics of tusavirus in fecal samples of patients with chronic diseases in Guangzhou, China

**DOI:** 10.3389/fmicb.2023.1205134

**Published:** 2023-06-15

**Authors:** Huan He, Yongzhi Li, Jiaqi Chen, Juxian Xian, Liting Zheng, Hengbiao Sun, Shunchang Fan, Jiaqi Fu, Qiushuang Li, Caiyun Chen, Minyi Liang, Minyi Zhang, Ruojun Wu, Gang Xiao, Qing Chen

**Affiliations:** ^1^Guangdong Provincial Key Laboratory of Tropical Disease Research, Department of Epidemiology, School of Public Health, Southern Medical University, Guangzhou, China; ^2^Clinical Laboratory, Third Affiliated Hospital of Southern Medical University, Guangzhou, China

**Keywords:** Tusavirus (TuV), chronic disease, genetic characteristics, protein structure prediction, B-cell epitopes, selection pressure

## Abstract

**Purpose:**

The Tunisian stool-associated parvovirus [Tusavirus (TuV)] is a novel member of the genus *Protoparvovirus,* which may be linked to diarrhea. Herein, we investigated the prevalence of TuV in different populations and analyzed its genetic and bioinformatic characteristics.

**Methods:**

This study was conducted in a tertiary hospital in Guangzhou (China) from February 2018 to July 2022. Demographic and clinical information and stool samples were collected from individuals who visited the hospital. ProtScale, SwissModel, Datamonkey, and other tools were used to analyze and predict the physicochemical parameters, tertiary structure, selection pressure, and B-cell epitopes of capsid viral protein 2 of TuV (VP2-TuV).

**Results:**

A total of 3,837 participants were enrolled, among which two stool samples from patients with chronic illnesses were tested positive for TuV DNA. However, no positive sample was detected among patients with diarrhea. Two near-complete genome sequences were amplified. The genetic analysis revealed the presence of diversity among TuVs isolated from distinct host species. Bioinformatics analysis revealed that VP2-TuV exhibited hydrophilic properties and lacked transmembrane domains and signal peptides. The secondary structure of VP2-TuV was composed mainly of random coils and β-strands. Selective-pressure analysis of the VP2 region suggested that TuV primarily underwent negative selection during evolution. Negatively selected codon sites coincided with residues comprising of B-cell epitopes, suggesting minimal changes in the immunogenicity of TuV over time.

**Conclusion:**

TuV was detected in patients with chronic diseases but not in patients with diarrhea. The putative roles of TuV in the pathogenicity of human diseases and zoonotic viruses must be determined by additional studies.

## 1. Introduction

The Tunisian stool-associated parvovirus [Tusavirus (TuV)] was first identified (through metagenomic studies) in a stool specimen from an 18-month-old child with unexplained diarrhea in Tunisia in 2014, implying a potential etiological relationship between the virus and the gastrointestinal illness affecting the patient (Phan et al., 2014). Since its initial discovery, TuV DNA has been identified in two adults living in Finland who presented with gastroenteritis (Mohanraj and Jokinen, 2021). Since then, there have been few reports of TuV, and its etiology and potential role in human diseases remains elusive. Recent detection of TuV in diarrheal or asymptomatic goats and sheep in Hungary suggests a plausible zoonotic origin for TuV infections (Reuter et al., 2022). Given the scarcity of published research on TuV DNA, further scrutiny of human specimens is warranted.

TuV is a member of the genus *Protoparovirus* of the family Parvoviridae, which comprises small nonenveloped DNA viruses with a single-stranded linear genome of approximately 5 kb. The TuV genome consists of two major open reading frames (ORFs) encoding the non-structural 1 (NS1) and the overlapping capsid viral proteins 1 and 2 (VP1 and VP2). NS1 encodes the viral helicase, while VP1 contains regions that are rich in glycine and phospholipase A2 (Phan et al., 2014). VP2, the primary capsid constituent, has the ability to self-assemble into virus-like particles (VLPs). These VLPs are frequently employed as antigens in serodiagnosis and contribute significantly to the virus pathogenicity and host immune response (Mietzsch et al., 2019). Mietzsch et al. compared the structure of TuV capsids with that of other parvoviruses. They cloned the VP2 protein of TuV (hereafter termed “VP2-TuV”) into the pFastBac1 plasmid to generate recombinant baculoviruses that express virus-like particles using the Bac-to-Bac system. Then, they utilized cryo-electron microscopy and image reconstruction to determine its capsid structure. The TuV capsid shares common features with other parvoviruses, including an eight-stranded anti-parallel β-barrel, depressions at the icosahedral 2-fold and surrounding the 5-fold axes, and a channel at the 5-fold axes. In addition, TuV might bind sialic acid (SIA) in a similar way to MVM (minute virus of mice) or CPV (canine parvovirus), which has been reported to be a receptor for infection and a hemagglutination receptor in some protoparvoviruses (Mietzsch et al., 2020).

In previous studies, TuV-DNA was detected in humans only in stool samples from patients who underwent diarrhea or fever in Tunisia and Finland. No TuV detection has been reported in China. Herein, we present evidence of TuV detection in several populations in Guangzhou, China, thereby expanding the geographic boundaries of TuV circulation and evaluating the characteristics of TuV-infected populations. Furthermore, we analyzed the genome sequences and structure of proteins to elucidate the molecular characteristics of TuV, which could provide a solid foundation for further exploration in viral analysis.

## 2. Materials and methods

### 2.1. Sample collection

We assessed patients with diarrhea who were treated as outpatients at the Third Affiliated Hospital of Southern Medical University in Guangzhou from February 2018 to December 2021. We also surveyed individuals who underwent health examinations during the same period. Furthermore, we recruited patients who were hospitalized between July 2021 and July 2022. Demographic and clinical information was collected for all survey participants. In addition, ~2 g of fresh feces was collected from each participant and placed in 1 mL of phosphate-buffered saline. The collected feces were stored at −80°C until use. Information on fecal characteristics [as determined by the Bristol Stool Scale (Lewis and Heaton, 1997)], such as occult blood, transferrin level, microscopic fungus, and microscopic fat droplets, was also acquired.

### 2.2. DNA extraction and polymerase chain reaction (PCR) amplification

After homogenization, fecal samples were centrifuged (8,000× *g*, 10 min at 4°C) to collect the supernatants. DNA was extracted from 200 μl of supernatant using the MiniBEST Viral RNA/DNA Extraction Kit (TaKaRa Biotechnology, Kusatsu, Japan) following the manufacturer protocols, and the extracted DNA was stored at −40°C. Samples were assayed for the presence of TuV DNA by a nested PCR targeting the partial NS1 region, using primers described previously (Phan et al., 2014). The PCR conditions were 95°C for 5 min, 35 cycles at 95°C for 30 s, 52°C or 51°C (for the first or second round, respectively) for 30 s, and 72°C for 1 min, and a final extension at 72°C for 10 min. Each PCR mixture had a total volume of 25 μl (12.5 μl of Green Master Mix (Promega, Fitchburg, WI, USA), 8.5 μl of sterilized H_2_O, 2 μl of DNA template, and 1.0 μl of each primer). In addition, BuV, CuV, and other enteroviruses, including human bocavirus, enterovirus, sapovirus, adenovirus, rotavirus, norovirus, and human astrovirus (Supplementary Table 1) (Noel et al., 1995; Häfliger et al., 1997; Jiang et al., 1999; Vennema et al., 2002; Harvala et al., 2008; Blinkova et al., 2009; Kumthip et al., 2019) were examined in TuV-positive samples to determine the presence of co-infection. Amplified products were detected by electrophoresis using 1.0% agarose gels and then verified by sequencing.

### 2.3. Amplification of the complete genome of TuV

Based on a reference sequence of TuV (GenBank accession number: KJ495710), 12 sets of primers (Supplementary Table 2) were designed to amplify the near-complete viral genome of positive samples. After all fragments had been sequenced, the full-length polyprotein sequences were assembled using Lasergene SeqMan (DNASTAR, Madison, WI, USA).

### 2.4. Phylogenetic analysis

The nucleotide (nt) sequences of TuV identified were compared with the corresponding sequences of other TuV strains and novel parvoviruses in GenBank using Basic Local Alignment (Supplementary Table 3).^1^ Alignment of multiple sequences was conducted using “CLUSTALW” within MEGA v7.0 (Oxford Molecular, Oxford, UK). Phylogenetic trees were generated by the neighbor-joining method with 1,000 bootstrap replications. Pairwise nucleotide(nt) and amino acid (AA) identities among all sequences were calculated in Bioinformatics Aider.^2^

### 2.5. Prediction of the basic properties of VP2-TuV

The basic physicochemical parameters of VP2-positive strains were analyzed by ProtParam^3^ and ProtScale.^4^ Prediction of transmembrane helices in proteins was analyzed by TMHMM v.2.0.^5^ The SignaIP 6.0^6^ was used to predict the signal peptide. NetPhos 3.1^7^ was employed to predict phosphorylation sites. Prediction of N-glycosylation sites was done by NetNGlyc 1.0.^8^

### 2.6. Prediction of the spatial structure of VP2-TuV

The secondary structure of VP2-TuV was predicted through PSIPRED.^9^ Models of the tertiary structure of VP2-TuV were built using SwissModel.^10^ To further verify the reliability of the prediction model, the Structure Analysis and Verification Server^11^ was used for evaluation.

### 2.7. Analysis of selection pressure and prediction of B-cell epitopes

Detection of the selection pressure on VP2-TuV was achieved using four methods [single likelihood ancestor counting (SLAC), fixed effects likelihood (FEL), mixed effects model of evolution (MEME), and fast unconstrained Bayesian approximation (FUBAR)] for inferring selection on Datamonkey.^12^ The B-cell epitopes of the capsid proteins of TuV were predicted using Sysbio.^13^

## 3. Results

### 3.1. Basic characteristics of participants

A total of 3,837 participants were enrolled during the study period and subsequently divided into six groups based on their health status: “diarrhea,” “non-diarrhea” (including premature infants and patients with orthopedic diseases), “physical examination,” “cancers,” “autoimmune diseases,” and “chronic diseases.” Demographic information of participants (age, sex, and disease type) across the six groups is presented in Table 1.

**Table 1:** Characteristics of groups used in the current study.

Groups	No. of individuals studied (*N* = 3,837)	Age range (median age, y)^a^	Sex^b^	Time of sample collection	Health status
Diarrhea	689	0–83 years (2)	Female, *N* = 268; Male, *N* = 421	2018 Feb – 2021 Dec	Acute gastroenteritis and/or gastrointestinal dysfunction
Non-diarrhea	506	0–22 years (1)	Female, *N* = 205; Male, *N* = 301	2018 Feb – 2021 Dec	Premature birth/ high risk/ low birth weight infant; acute fracture or congenital deformity of finger
Physical examination	419	0–86 years (46)	Female, *N* = 251; Male, *N* = 168	2018 Jul - 2021 Dec	Without diarrhea; healthy
Cancers	533	5–90 years (57)	Female, *N* = 261; Male, *N* = 272	2021 Jul - 2022 Jul	Malignant tumor; malignant lymphoma; leukemia and multiple myeloma
Autoimmune diseases	604	10–94 years (48)	Female, *N* = 397; Male, *N* = 207	2021 Jul - 2022 Jul	Autoimmune systemic disease, autoimmune organ specific disease,
Chronic diseases	1,086	20–95 years (64)	Female, *N* = 494; Male, *N* = 592	2021 Jul - 2022 Jul	Chronic kidney disease, inflammatory bowel disease, autoinflammatory disease, neuromyelitis optica spectrum disease, hypertension, diabetes, coronary heart disease, ischemic stroke, chronic obstructive pulmonary disease

a y: year.

b F: female; M: male.

### 3.2. TuV-positive patients

Of the 3,837 stool samples analyzed, only two were positive for TuV. PCR products were analyzed by agarose gel electrophoresis, showing expected sizes of approximately 247 bp in positive samples (Supplementary Figure 1). These were designated “GZ814” and “GZ1068,” and both belonged to the “chronic diseases” group. GZ814 was from a 48-year-old woman with a tension headache who underwent postoperative radiotherapy for nasopharyngeal carcinoma in 2002. GZ1068 was from a 58-year-old man with type-2 diabetes mellitus (T2DM) and hypertension (Table 2). Bristol Stool Scale analysis showed no signs of diarrhea in any of the samples except for the presence of fat droplets in the stool of GZ814, and none of the patients had a fever. Laboratory test results did not indicate any correlation between these two TuV-positive patients (Supplementary Table 4), and no other enteroviruses were detected in their samples (Table 2).

**Table 2:** Samples obtained from patients that tested positive for Tusavirus DNA.

Sample	Sex^a^	Age^b^	Date of sample collection (dd/mm/yyyy)	Case history	Disease	Symptom	Other viruses^c^
GZ814	F	58y	01/12/2021	Postoperative radiotherapy for nasopharyngeal carcinoma (2002)	Tension headache	Headache, hoarseness, xerostomia	All negative
GZ1068	M	48y	11/01/2022	Hypertension	Type 2 diabetes mellitus; Hypertension	Xerostomia, polydipsia, polyuria, nausea, lumbago, blurred vision, acroanesthesia	All negative

a F: female; M: male.

b y: year.

c Other viruses included bufavirus, cutavirus, human bocavirus, enterovirus, sapovirus, adenovirus, rotavirus, norovirus, and human astrovirus.

### 3.3. Characteristics and phylogenetic analyses of TuV

The near-complete sequences of the two TuV-positive strains (GZ814 and GZ1068) were amplified and deposited in GenBank (accession numbers: OQ708493 and OQ708494). GZ814 and GZ1068 were 4,424 nt and 4,428 nt in length, respectively, with a partial 5′ untranslated region (UTR) of 244 nt, complete NS1 (625 aa) replicase and complete VP1 open reading frame (715 aa) containing VP2 (565 aa), and a partial 3’ UTR (69–73 nt). BLASTn analysis showed that nt and AA similarities of the genomic regions and proteins of NS1, VP1, and VP2 between GZ814 and GZ1068 were 98.88 to 99.12% and 99.36 to 99.82% compared to each other. In comparison with the reference strain KJ495710, the similarity of complete sequences was 95.04–95.12%, with 93.23–93.49%, 97.02–97.20%, and 97.05–97.29% nt identities, and 98.72%, 99.58–99.86%, and 99.65–99.82% AA identities at NS1, VP1, and VP2, respectively (Table 3). In contrast to KJ495710, the positive samples in our study included two AA deletions in the undefined area between NS1 and VP1. Multiple-sequence alignment of the VP2 sequences of TuV from different species by CLUSTALW analysis was shown in Supplementary Figure 2. Compared with the sequences from humans, there were 53 variants in AA residues of VP2 sequences from goats with gastroenteritis and 4 from asymptomatic sheep. In addition, we conducted a comparative sequence analysis for each functional region between our sequences and other parvoviruses in GenBank from animals (Table 4). The GZ814 and GZ1068 shared 94.57–94.73, 89.80%, and 89.93–90.11% AA identities with the goat TuV at the NS1, VP1, and VP2 regions, respectively.

**Table 3:** Nucleotide and putative amino acid sequence identity of the NS1, VP1 and VP2 regions between GZ814/GZ1068 and other Protoparvovirus reference sequences from human^a^.

Gene region^b^	Human				
	Tusavirus	Cutavirus	Bufavirus 1	Bufavirus 2	Bufavirus 3
GZ814					
NS1	93.23/98.72	27.47/6.82	27.47/6.70	27.56/7.57	27.16/7.27
VP1	97.20/99.58	30.48/7.26	29.12/6.84	30.71/8.38	30.66/7.26
VP2	97.29/99.65	27.71/5.61	28.81/5.78	27.68/5.43	27.18/6.98
GZ1068					
NS1	93.49/98.72	27.21/6.82	27.12/6.70	27.26/7.57	26.87/7.27
VP1	97.02/99.86	30.38/7.26	29.36/6.84	30.52/8.38	30.52/7.26
VP2	97.05/99.82	27.42/5.61	28.51/5.78	27.33/5.43	27.07/6.98

Values were described as nucleotide identity (%) /amino acid identity (%).

a Reference sequences include: Human tusavirus (accession no. KJ495710), Human cutavirus (accession no. NC039050), Human bufavirus 1 (accession no. JQ918261), Human bufavirus 2 (accession no. JX027297), and Human bufavirus 3 (accession no. AB847987).

b NS: non-structural; VP: viral protein.

**Table 4:** Nucleotide and putative amino acid sequence identity of the NS1, VP1 and VP2 regions between GZ814/GZ1068 and other Protoparvovirus reference sequences from animals^a^.

Gene region^b^	Goat	Canine		Porcine	Megabat	Feline	Mink	Rodent		
	Tusavirus	Parvovirus	Bufavirus	Parvovirus	Bufavirus	Parvovirus	Parvovirus	Rat bufavirus	Hamster parvovirus	Mice parvovirus
GZ814										
NS1	84.16/94.73	33.93/13.75	29.24/6.89	28.85/6.79	30.22/10.11	33.93/13.75	33.83/13.75	30.12/6.82	32.04/14.71	32.24/14.71
VP1	81.83/89.80	32.90/13.27	33.10/13.58	28.67/5.83	29.12/7.70	26.87/6.93	26.87/6.93	28.78/7.65	27.51/5.75	29.39/7.57
VP2	81.65/90.11	26.71/5.98	30.34/6.85	28.79/7.41	26.96/5.86	26.88/6.15	26.77/6.15	26.44/5.46	27.48/7.14	25.71/5.27
GZ1068										
NS1	84.11/94.57	33.63/13.75	29.24/6.89	28.90/6.79	30.17/10.11	33.63/13.75	33.53/13.75	29.71/6.82	31.89/14.71	32.19/14.71
VP1	82.06/89.80	32.90/13.27	33.20/13.72	28.62/5.83	29.16/7.57	26.78/6.79	26.78/6.79	29.01/7.51	27.47/5.62	29.44/7.43
VP2	81.71/89.93	26.83/5.98	30.22/6.85	28.67/7.41	27.07/5.86	27.00/6.15	26.88/6.15	26.55/5.46	27.43/7.31	25.88/5.44

Values were described as nucleotide identity (%) /amino acid identity (%).

a Reference sequences include: Goat tusavirus (accession no. OL692339); Canine parvovirus (accession no. JN867610); Canine bufavirus (accession no.MT542983); Porcine parvovirus (accession no. U44978); Megabat bufavirus (accession no.LC085675); Feline parvovirus (accession no. EU659111); Mink parvovirus (accession no. D00765); Rat bufavirus (accession no. KT716186); Hamster parvovirus (accession no. U34255); Mice parvovirus (accession no. J02275).

b NS: non-structural; VP: viral protein.

**Figure 1:**
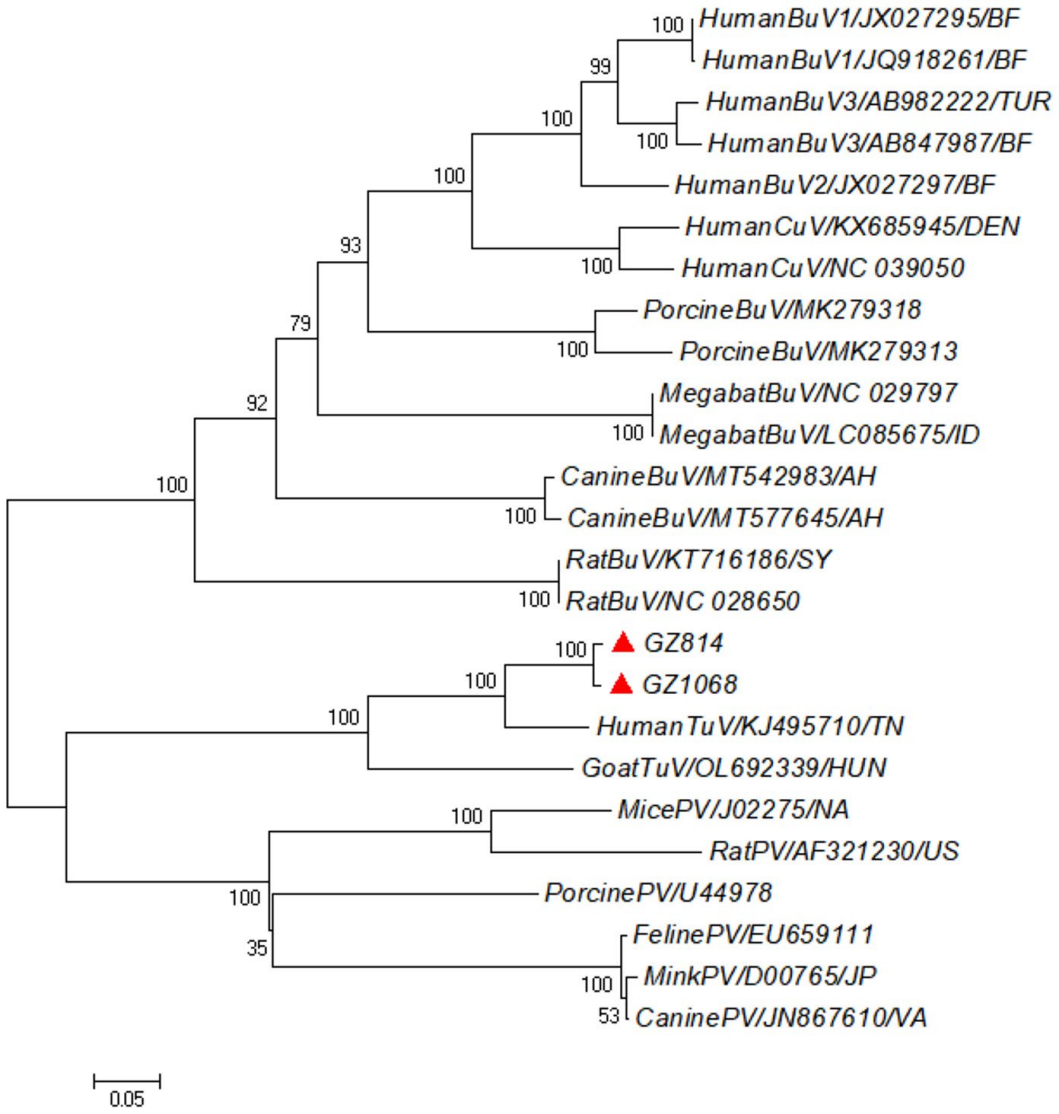
Phylogenetic tree based on the near-complete genome sequences of HumanTuV and other novel parvoviruses. The neighbor-joining method with the Nei-Gojobori (Jukes-Cantor) substitution model was used for the construction, with 1,000 bootstrapping replicated to test the branching. Red triangles indicated the Human TuV sequences identified in this study. TuV, tusavirus; BuV, bufavirus; CuV, cutavirus; PV, parvovirus.

Phylogenetic analysis was performed using the neighbor-joining method on the near-complete genome sequences of parvovirus strains obtained from both humans and animals. The two positive strains in our study were very closely related to each other and clustered to the human TuV (GenBank accession number: KJ495710) (Figure 1). Then, these strains clustered together with another TuV (GenBank accession number: OL692339) from a goat with gastroenteritis. Notably, all TuV sequences formed a separate group alongside parvovirus strains from various animal species.

### 3.4. Physicochemical properties of proteins

Analysis by ProtParam showed that the VP2 proteins of GZ814 and GZ1068 consisted of 565 AAs. The top-three AAs with the highest proportion were Thr (10.4%), Asn (8.0%), and Gly (8.0%) (Supplementary Table 5). Among the 565 AAs, there were 52 negatively charged AA residues (Asp+Glu) and 38 positively charged AA residues (Arg + Lys). The formulas of GZ814 and GZ1068 were C_2783_H_4222_N_770_O_854_S_16_ and C_2784_H_4224_N_770_O_854_S_16_, with molecular weights of 62643.74 Da and 62657.77 Da, respectively. The instability index of VP2 was >40.00, indicating that the protein was stable.

The profiles of the hydrophilic and hydrophobic sequences of VP2-TuV were obtained using ProtScale according to its default algorithm. The grand average of hydropathicity (GRAVY) was −0.476 for GZ814 and − 0.475 for GZ1068, suggesting that VP2-TuV was a hydrophilic protein. Taking GZ1068 as a template, VP2-TuV had four high-scoring (>1.5) peaks located at positions 158, 227, 530, and 532–533, of which the maximum value was 2.333 at position 530. The lowest peak was observed at 369–370, with a minimum score of −2.922. Analysis of VP2-TuV using ProtScale revealed that of the 557 (position 5–561) AAs, 72.89% (406) were distributed in the low-scoring (<0) area, while 27.11% (151) were in the high-scoring (>0) area. These data indicated that VP2-TuV had many hydrophilic domains and was a hydrophilic protein (Supplementary Figure 3).

According to TMHMM, VP2-TuV was completely outside the membrane and lacked a transmembrane domain. Signal P was used to analyze the signal peptide of VP2-TuV. The predicted value was 0.0005, and a signal peptide was not present, indicating that it was a non-secretory protein.

When the threshold for potential phosphorylation sites was 0.5, there were 67 potential phosphorylation sites in VP2-TuV (Supplementary Table 6): 28 Ser sites, 33 Thr sites, and six Tyr sites (Supplementary Figure 4). VP2-TuV included eight probable N-glycosylation sites (N31, N48, N70, N85, N131, N217, N377, N486) (Supplementary Figure 5).

### 3.5. Predictions of the secondary and tertiary structures of VP2-TuV

**Figure 2:**
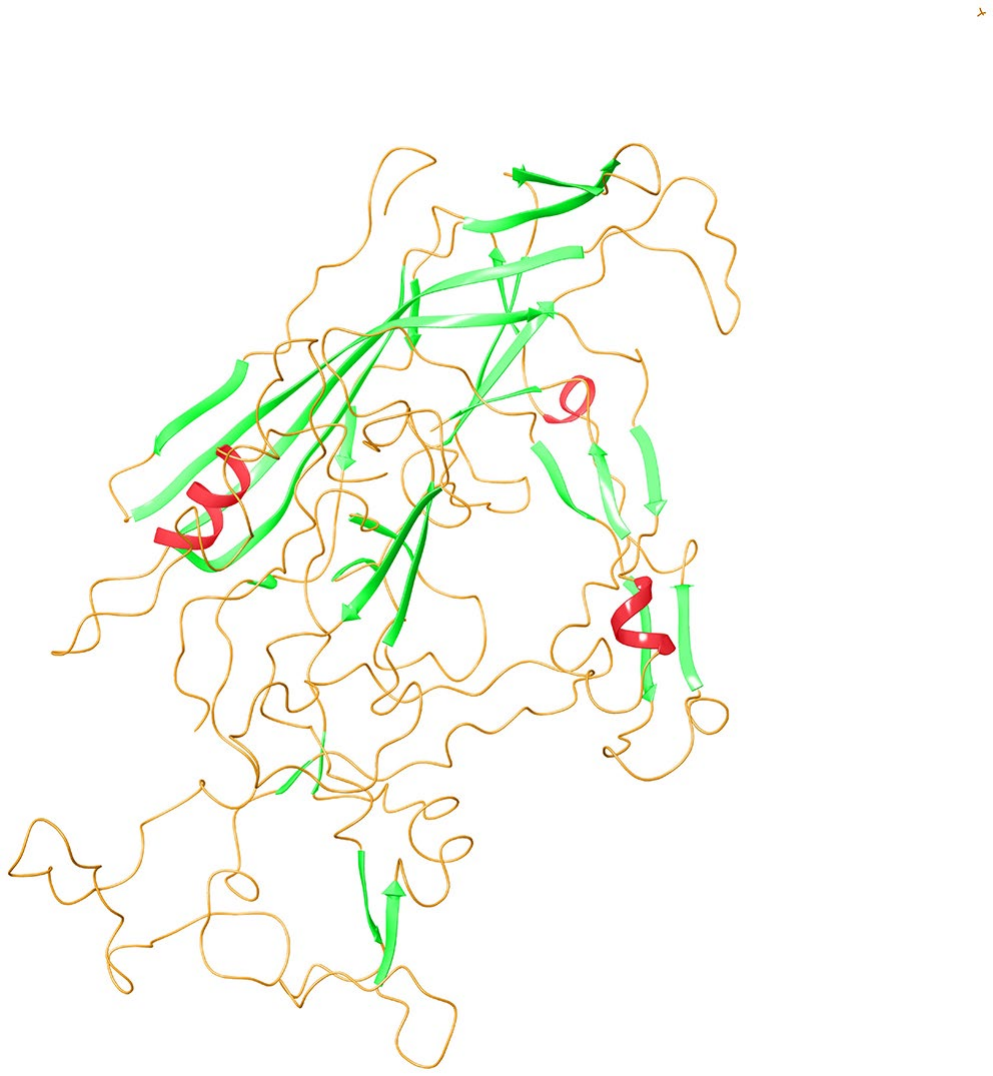
Tertiary structure prediction of VP2 protein of GZ1068. The red wavy lines represented the α-helices; the green sheets indicated β-strands; the orange curves represented random coils. VP, viral protein.

The secondary structure prediction of VP2-TuV revealed the presence of 2 α-helices, 27 β-strands, and 30 random coils. Small nonpolar AAs and polar AAs comprised 38.30 and 31.21% of VP2-TuV, respectively, while hydrophobic amino acids accounted for 19.86%. Consequently, the secondary structure of VP2-TuV comprised strands and random coils. To create the tertiary structure of VP2-TuV from GZ1068, the deposited capsid structure (Protein Database ID: 6X2K) was utilized as a template (Figure 2). The predicted tertiary structure consisted primarily of strands and random coils, in line with the secondary structure. The model exhibited a high degree of similarity to the template, with a calculated value of 98.90%. Moreover, the model obtained a high-quality score, with a QMEAN (Qualitative Model Energy ANalysis) score of 0.890. Finally, the Ramachandran plot showed that >90% of amino acid residues were located in the red and yellow regions, indicating a stable protein structure, with all amino acids possessing suitable dihedral angles (Supplementary Figure 6). The SwissModel prediction of VP2-TuV from GZ1068 in our study was stable and trustworthy.

### 3.6. Analysis of selection pressure and prediction of B-cell epitopes based on VP2-TuV

**Figure 3:**
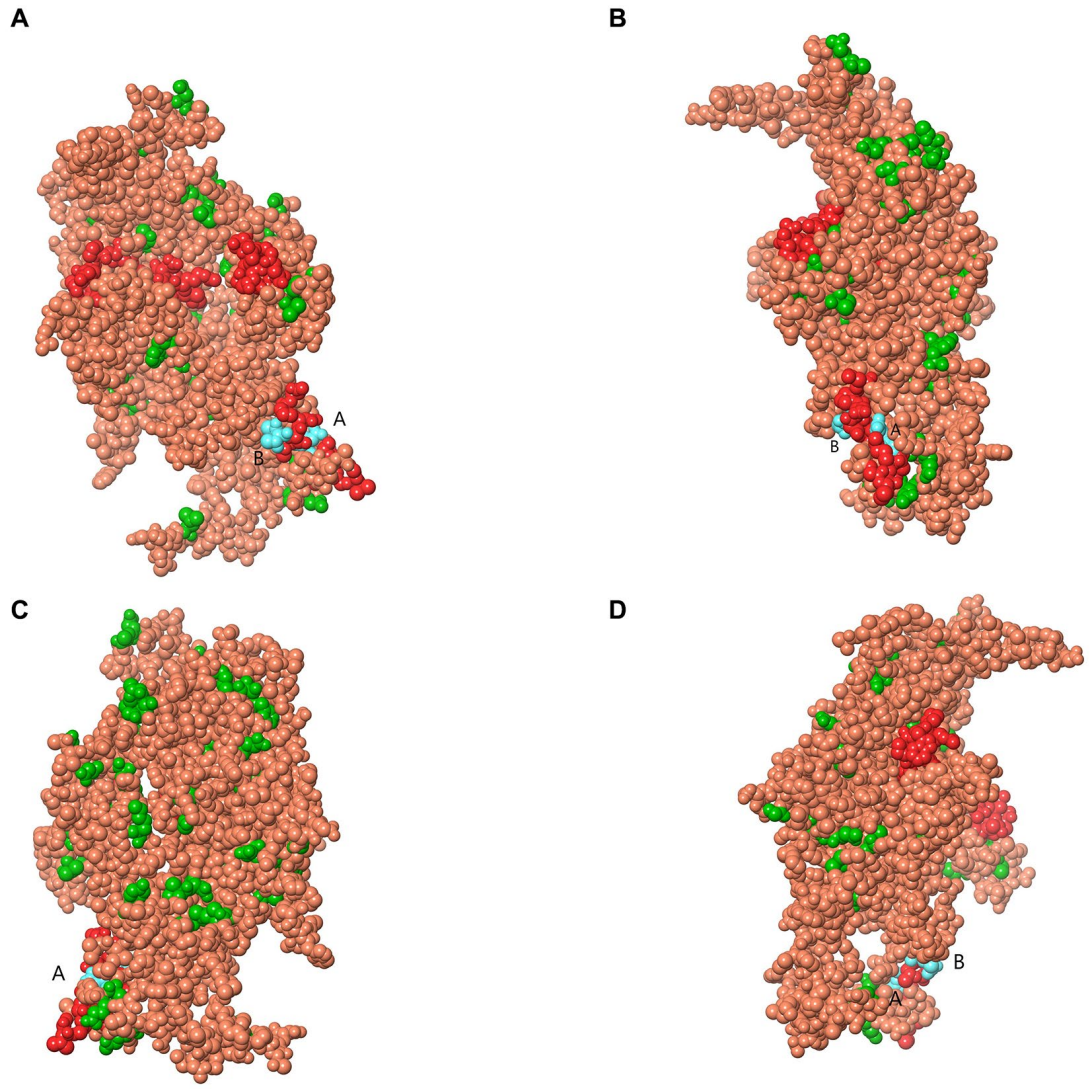
Map of B-cell epitopes prediction and selection pressure site of VP2-TuV gene **(A–D)**. The predicted B-cell epitopes in the VP2 protein was indicated in red. The selective pressure sites were indicated in green. Codons 415 and 416 **(A)**, 418, and 419 **(B)** were located on the predicted B-cell epitopes, indicated in blue. TuV, tusavirus; VP, viral protein.

Analysis of selection pressure of all VP2-TuV strains revealed 54 negative sites, of which 52 were detected by several methods (Supplementary Table 7). To further comprehend the effect of selection pressure on the immune response against TuV, we analyzed VP2 sequences in B-cell epitopes. The VP2-dominant epitopes of GZ814 and GZ1068 strains were located on AA residues 63–82, 169–188, and 408–427 (Table 5). It is noteworthy that α-helices, β-strands, or hydrophobic regions are not typically capable of forming epitopes; therefore, the B-cell epitopes were ultimately identified as being located at AA residues 68–75, 173–188, and 410–423. Interestingly, a thorough analysis of pressure-selected B-cell epitopes revealed that codons 415, 416, 418, and 419 were located on the predicted B-cell epitopes (Figure 3).

**Table 5:** The results of B-cell epitope prediction.

Strains	Location	Epitope	Score
GZ814/GZ1068	63–82	YKIIPTQNNTAVQTVGHMMD	1.000
	169–188	PYTPAAIRSETLGFYPWRPT	0.932
	408–427	TAGVGKNGETATSDPNLVRY	0.877

## 4. Discussion

This was the first report on the presence of TuV in human stool samples in China. Two TuV-positive samples (designated GZ814 and GZ1068) were obtained from adults diagnosed with tension nasopharyngeal carcinoma and T2DM with hypertension, respectively. Previous studies have shown TuV DNA detection in fecal specimens obtained from individuals who exhibited severe clinical signs of diarrhea or gastroenteritis (Phan et al., 2014; Mohanraj and Jokinen, 2021). However, none of the TuV-positive specimens in our study was associated with fever or symptoms of severe gastroenteritis, and the stool laboratory test results of these cases showed no significant abnormalities. Moreover, positive samples were not observed in the diarrhea group in our study. There is still uncertainty about the role of TuV in acute gastroenteritis and other illnesses. We inferred that TuV infection might not be strongly linked to diarrhea. However, more pathogenicity studies are required to clarify whether TuV infection is a significant contributor to gastrointestinal disorders. Recently, TuV was detected in stool samples from goats and sheep in Hungary using screening primers for VP1, and the complete genome sequence of TuV was obtained, indicating the possibility of domestic and/or wild animals being reservoirs and hosts for TuV (Reuter et al., 2022).

Only two TuV genomes with near-complete coding sequences were available in GenBank. One genome (GenBank accession number: KJ495710) was amplified and sequenced in the initial discovery from human diarrheal feces. The other genome (GenBank accession number: OL692339) originated from the feces of a goat with diarrhea. Herein, we acquired almost the complete gene sequences from both positive samples (4424–4428 nt). The complete gene sequences acquired in this study could provide more comprehensive information on the genetic characteristics of TuV prevalent in China. Based on the blastn analysis, the sequences demonstrated a higher similarity to the human TuV strain (KJ495710) than goat TuV strain (OL692339), indicating that TuV exhibits genetic diversity across various host species.

According to a comparison of capsid sequences and structures, scholars have postulated TuV to be a “hybrid virus” with molecular properties falling between those of primate and non-primate protoparvoviruses (Mietzsch et al., 2020). Additionally, a metagenomics study of fur seals in Brazil revealed some sequences that shared 39–82% of their AA composition with that of human TuV (Kluge et al., 2016). Moreover, based on the binding pattern of its sialic acid (SIA) cell receptor, TuV has been shown to display greater affinity to a wider range of glycans than human CuV (Mohanraj and Jokinen, 2021). Meanwhile, in our study, phylogenetic analysis based on the near-complete sequencing showed that the TuV sequence shared a common root with parvovirus identified in animals but had a more distant relationship with human parvovirus, supporting that the TuV might have a wide range of hosts (Reuter et al., 2022). Collectively, these findings warrant further studies in different hosts to explore the possibility that TuV might be a zoonotic virus.

In parvoviruses, VP2 is the main component of capsid proteins, which has been identified as an inducer of neutralizing antibodies, thereby enabling the determination of parvovirus antigenicity (Hashemzadeh et al., 2017; Mietzsch et al., 2020). Comparison of nt and AA homology revealed that the nt sequence of VP2 in TuV shared a homology range of 81.65–97.29% across humans and goats, while its AA homology was between 89.93 and 99.82%. These findings suggest that VP2 of TuV exhibited mutagenicity during evolution and differs across various host species. Subsequently, we utilized bioinformatics information of VP2-TuV to predict the physical and chemical properties, structure, and function of proteins to gain insights into the protein information of VP2-TuV. Analysis of the physicochemical properties of VP2 revealed it to be a hydrophilic protein encoding 565 AAs, and there was neither a transmembrane domain nor a signal peptide in this region. These insights indicated that VP2 was synthesized mainly in the endoplasmic reticulum of host cells and was not secreted out of cells after synthesis but was directly employed in TuV assembly (Fujishima et al., 2011). Glycosylation and phosphorylation are the most important post-translational modifications *in vivo*. Glycosylation has an important effect on the stability and localization of the folding of proteins. We discovered 67 potential phosphorylation sites and 8 potential N-glycosylation sites in VP2-TuV, suggesting its involvement in transcriptional regulation through phosphorylation.

We demonstrated that the VP2-TuV protein was largely composed of β-strands and random coils, with a small proportion of α-helices. The α-helix and β-strand regions of a protein are the main structural elements that provide stability to the molecule (Ghozlane et al., 2009). Random crimping is the flexible region of a protein and a possible site for antigen epitopes. Despite the high proportion of β-strands in VP2-TuV, the random coils distributed on the surface, combined with its hydrophilic characteristics, facilitated the binding of VP2-TuV to other structural proteins on the cell membrane, thereby providing favorable conditions for assembly of the TuV membrane.

Selection pressure on specific AA sites in proteins is thought to be part of adaptive evolution (Miller et al., 2009). To understand the impact of external selection pressure on immune response to the virus, we selected the TuV-VP2 sequence (which has more AA mutations than in NS1) for analysis of selection pressure using SLAC/FEL/FUBAR/MEME methods, with the aim of exploring whether external selection pressure was related to these mutations. Our analysis revealed 54 negatively selected sites and no positively selected sites, indicating a tendency toward sequence conservation rather than increased genetic variation resulting from past evolutionary pressures. Therefore, our findings suggested that external selection pressure may not have significantly contributed to the presence of mutations.

To gain a more comprehensive understanding of the antigenic sites of TuV, we conducted an analysis of the B-cell epitopes within the VP2 sequences. A B-cell epitope comprises a set of AA residues of an antigen that make direct contact with the residues belonging to an antibody (Sivalingam and Shepherd, 2012). These residues can be recognized by specific B-cell receptors or specific antibody molecules of the immune system, thereby determining the immunogenicity of a virus (Huang et al., 2008). After eliminating regions where epitopes were not readily formed (such as α-helix, β-strand, hydrophobic regions), three B-cell epitopes were identified within VP2-TuV: 68–75, 173–188, and 410–423. Furthermore, negatively selected codon sites 415, 416, 418, and 419 were located on the predicted B-cell epitopes, indicating that selection pressure did not significantly alter TuV’s B-cell epitopes. As a result, the conservation of epitope residues suggests that the immunogenicity of TuV may not change considerably, making the production of new TuV serotypes difficult, which could help in vaccine development and the prevention of viral propagation.

Our study had one main limitation. TuV DNA was detected exclusively in human fecal samples and not in serum or nasopharyngeal swabs.

## 5. Conclusion

Although TuV is considered as a diarrhea-associated virus, it was not detected in people with diarrhea in this study. Instead, it was found in individuals with chronic diseases, among which one had nasopharyngeal carcinoma and the other had T2DM and hypertension. The near-complete genomes of GZ814 and GZ1068 were sequenced. Analysis of the genetic characteristics indicated the existence of diversity among TuVs from different host species and showed that the immunogenicity of TuV may not be affected considerably. The putative roles of TuV in the pathogenicity of human diseases and zoonotic viruses warrant further studies.

## Author’s note

All named authors in this article meet the International Committee of Medical Journal Editors (ICMJE) criteria for authorship, take responsibility for the integrity of the work as a whole, and have given their approval for this version to be published.

## Data availability statement

The datasets presented in this study can be found in online repositories. The names of the repository/repositories and accession number(s) can be found in the article/Supplementary material.

## Ethics statement

The studies involving human participants were reviewed and approved by Ethics Committee of Southern Medical University (2019001). Oral informed consent to participate in this study was provided by the participants’ legal guardian/next of kin.

## Author contributions

HH and YL: methodology, investigation, formal analysis, and writing-original draft preparation. JC, JX, and LZ: formal analysis. HS and SF: data curation. JF, QL, CC, and ML: investigation. MZ and RW: data curation and methodology. GX and QC: project administration, conceptualization, funding acquisition, supervision, and writing – review and editing. All authors contributed to the article and approved the submitted version.

## Funding

This work was supported by the National Natural Science Foundation of China (82273697).

## Conflict of interest

The authors declare that the research was conducted in the absence of any commercial or financial relationships that could be construed as a potential conflict of interest.

## Publisher’s note

All claims expressed in this article are solely those of the authors and do not necessarily represent those of their affiliated organizations, or those of the publisher, the editors and the reviewers. Any product that may be evaluated in this article, or claim that may be made by its manufacturer, is not guaranteed or endorsed by the publisher.

## Footnotes

^1^ www.ncbi.nlm.nih.gov

^2^ https://github.com/ZhijianZhou01/BioAider/releases

^3^ https://web.expasy.org/protparam

^4^ https://web.expasy.org/protscale

^5^ http://www.cbs.dtu.dk/services/TMHMM

^6^ https://www.healthtech.dtu.dk/

^7^ https://services.healthtech.dtu.dk/service.php?NetPhos-3.1

^8^ https://services.healthtech.dtu.dk/service.php?NetNGlyc-1.0

^9^ http://bioinf.cs.ucl.ac.uk/psipred

^10^ https://swissmodel.expasy.org

^11^ http://services.mbi.ucla.edu/SAVES

^12^ www.datamonkey.org

^13^ http://sysbio.unl.edu/SVMTriP/prediction.php
