# Supplementary material for: Identification and genetic characteristics of tusavirus in fecal samples of patients with chronic diseases in Guangzhou, China
Source: Front Microbiol. 2023 Jun 15;14:1205134. doi: 10.3389/fmicb.2023.1205134 (PMC10310535; doi:10.3389/fmicb.2023.1205134)

**Supplementary Table 1**. Primers used for sequences amplification of other enteroviruses^a^.

| **Virus and oligonucleotide**^b^ | **Region**^c^ | **Nucleotide sequence 5'-3'**^d^ | **Amplicon (bp)** |
| --- | --- | --- | --- |
| *Bufavius* | *NS1* |  | 440 |
| BF-F1 |  | TCAACAATCACTCAGGCAAATGG |  |
| BF-R1 |  | AGTTTGCCTGGATGTTCTTTGA |  |
| BF-F2 |  | CTAACACTGGTACTTGCTATGGAC |  |
| BF-R2 |  | TTCTCTGGTGATGATTCTTTTGTC |  |
| *Cutavirus* | *VP1* |  | 427 |
| CutaV-F1 |  | CAAACTACCAACTTACTGCTACCA |  |
| CutaV-R1 |  | GTTAGTCTGGTTCCTTCAGTTGTTG |  |
| CutaV-F2 |  | GAATACAATAGACATAAACCAAGCAGAC |  |
| CutaV-R2 |  | TGCTTGTGAAAATGAACTGCCTG |  |
| *Human bocavirus* | *VP1* |  | 455 |
| Boca-F1 |  | CGCCGTGGCTCCTGCTCT |  |
| Boca-R1 |  | TGTTCGCCATCACAAAAGATGTG |  |
| Boca-F2 |  | GGCTCCTGCTCTAGGAAATAAAGAG |  |
| Boca-R2 |  | CCTGCTGTTAGGTCGTTGTTGTATGT |  |
| *Enterovirus* | *5’ UTR* |  | 400 |
| EV06-F1 |  | CAAGCACTTCTGTTTCCCCGG |  |
| EV03-R1 |  | ATTGTCACCATAAGCAGCCA |  |
| EV06-F2 |  | CAAGCACTTCTGTTTCCCCGG |  |
| EV05-R2 |  | CACGGACACCCAAAGTA |  |
| *Sapovirus* | *3D* |  | 469 |
| Sap36-F |  | GTTGCTGTTGGCCATTAACA |  |
| Sap35-R |  | GCAGTGGGTTTGAGACCAAAG |  |
| *Adenovirus* | *hexon gene* |  | 482 |
| Ad1-F |  | TTCCCCATGGCTCAYAACAC |  |
| Ad2-R |  | CCCTGGTAKCCRATRTTGTA |  |
| *Rotavirus A* | *VP7 gene* |  | 346 |
| RoV2-F1 |  | CTTTAAAAGAGAGAATTTCCCTCTG |  |
| RoV1-R1 |  | GTCACATCATACAATTCTAATCTAAG |  |
| RoV3-F2 |  | TGTATGGTATTGAATATACCAC |  |
| RoV4-R2 |  | ACTCATCCTGTTGGCCAWCC |  |
| *Norovirus Ⅰ* | *RNA polymerase* |  | 187 |
| JV12Y-F1 |  | ATACCACTATGATGCAGAYTA |  |
| JV13I-R1 |  | TCATCATCACCATAGAAIGAG |  |
| G1-F2 |  | TCNGAAATGGATGTTGG |  |
| JV13I-R2 |  | TCATCATCACCATAGAAIGAG |  |
| *Norovirus Ⅱ* | *RNA polymerase* |  | 236 |
| JV12Y-F1 |  | ATACCACTATGATGCAGAYTA |  |
| JV13I-R1 |  | TCATCATCACCATAGAAIGAG |  |
| JV12Y-F2 |  | ATACCACTATGATGCAGAYTA |  |
| NoroII-R2 |  | AGCCAGTGGGCGATGGAATTC |  |
| *Human astrovirus* | *ORF2* |  | 449 |
| Mon269-F |  | CAACTCAGGAAACAGGGTGT |  |
| Mon270-R |  | TCAGATGCATTGTCATTGGT |  |
| *Human Parechovirus* | *VP3* |  | 304 |
| HPeV-F1 |  | GAYAATGCYATMTAYACWATYTGTGA |  |
| HPeV-R1 |  | ACWGTRAARATRTCHACATTSATDG |  |
| HPeV-F2 |  | TTYTCMACHTGGATGMGGAARAC |  |
| HPeV-R2 |  | DGGYCCATCATCYTGWGCTGA |  |

^a^Primers for enteroviruses were published previously, which have been cited in the manuscript. Genome locations of the primers are based on the sequence of Human bufavirus 1 (accession no. JQ918261), Human cutavirus (accession no. NC039050), Poliovirus type Sabin 3 (accession no. X00596), Norwalk virus (accession no. M87661.2), Adenovirus (accession no. X76549), Human Wa rotavirus (accession no. KO2033) and Echovirus (accession no. L02971);

^b^F: forward; R: reverse;

^c^NS: non-structural; VP: viral protein; UTR:untranslated Region; ORF: open reading frame;

^d^Mixed bases in degenerated primers: W = A/T, Y = C/T, K = G/T, R = A/G.

**Supplementary Table 2**. Primers used for near-complete genome sequences amplification of TuV^a^ based on the previously published reference^b^.

| **Primer**  **name^c^** | **Nucleotide sequence(5′-3′)** | **Amplicon (bp)** |
| --- | --- | --- |
| TuV-01-F | CCTGTCTAAAAAAGGCAAAAAAG | 475 |
| TuV-01-R1 | CTTGATCTTTACTCCATTCTGCT |  |
| TuV-01-R2 | TCCTTCGTCTCTGTTAGTGAT |  |
|  |  |  |
| TuV-02-F | GGACATCACTAACAGAGACGA | 514 |
| TuV-02-R1 | AAGCATCCAATCTTCTGCAGT |  |
| TuV-02-R2 | TTGCTCACTGCCAACCGTT |  |
|  |  |  |
| TuV-03-F1 | GAGAAAAGATGCTTGGTGGCT | 387 |
| TuV-03-R1 | TCCTGTTGTAGCTGGTCCATA |  |
| TuV-03-F2 | ACGGTTGGCAGTGAGCAAG |  |
| TuV-03-R2 | GTTTAGGCAGCACATTATAGCAT |  |
|  |  |  |
| TuV-04-F | AGCATACAGAGCCATTAGAGA | 377 |
| TuV-04-R1 | GTCTCCTGGTAACTTGGTTGT |  |
| TuV-04-R2 | CTCCGACTTGAGTTATGTCCT |  |
|  |  |  |
| TuV-05-F | GGACTCATTCCCACTGAAGA | 353 |
| TuV-05-R1 | GTCCAAGTCGTTGCCTGGT |  |
| TuV-05-R2 | CCATAAGGTTTGGTGCTGAC |  |
|  |  |  |
| TuV-06-F | GAGAAAGACGAGCTGTTAGAC | 458 |
| TuV-06-R1 | CACCTCCTCCTGAAGATGAA |  |
| TuV-06-R2 | GATATGTGCAGGTGCAGGT |  |
|  |  |  |
| TuV-07-F1 | CCTAGACCCATACTGGAACT | 381 |
| TuV-07-R1 | CTAGAGAGTCTGGTCTTGTGA |  |
| TuV-07-F2 | GGAGACTAGGACACTGGAT |  |
| TuV-07-R2 | TGCACGTAATCGTGGCTGTA |  |
|  |  |  |
| TuV-08-F | GCACAGCAACCAGTCAGAG | 463 |
| TuV-08-R1 | CACTACAGTAGGTCTCCATG |  |
| TuV-08-R2 | CATCATTACTGCAGTGAGGTC |  |
|  |  |  |
| TuV-09-F | GATGATGACAACCACACCCA | 444 |
| TuV-09-R1 | CTGGTAGTTTGCCAATGTCTG |  |
| TuV-09-R2 | CAATTGGAAGCTGCGTCTCA |  |
|  |  |  |
| TuV-10-F | CAGCATCATCAACCACAGCT | 645 |
| TuV-10-R1 | GTATCTGACTAGGTTTGGATCA |  |
| TuV-10-R2 | CAGTTGCTTGGTATGGATGTT |  |
|  |  |  |
| TuV-11-F | CACACACACACTTGAAGGAC | 589 |
| TuV-11-R1 | TTGGTGAGGAGGTCTAAGCTT |  |
| TuV-11-R2 | GGATCTGTTTGTCCCATATTTG |  |
|  |  |  |
| TuV-12-F1 | CTGCAGGAGTTGGTAAGAATG | 416 |
| TuV-12-F2 | ACCTGTTTACCCACATGGTCA |  |
| TuV-12-R | GTTGGTTGCTTTATAGAAAAGAG |  |

^a^TuV: Tusavirus;

^b^Reference: Huamn tusavirus (accession no. KJ495710);

^c^F: forward; R: reverse

**Supplementary Table 3**. General information and GenBank accession numbers of reference strains used in this study.

| **Strains** | **GenBank accseeion no.** | **Organism** | **Country**^a^ | **Nucleotides(bp)** | **Host** |
| --- | --- | --- | --- | --- | --- |
| BF.7 | JX027295 | Bufavirus-1 | BF | 4822 | Human |
| BF.96 | JQ918261 | Bufavirus-1 | BF | 4912 | Human |
| BF.39 | JX027297 | Bufavirus-2 | BF | 4562 | Human |
| BTN-63 | AB847987 | Bufavirus-3 | BT | 4733 | Human |
| AHP-740 | AB982222 | Bufavirus-3 | TUR | 4745 | Human |
| CGG5-268 | KX685945 | Cutavirus | DEN | 4452 | Human |
| BR-337 | NC_039050 | Cutavirus | BR | 4456 | Human |
| KT-G5 | OL692339 | Tusavirus | HU | 4316 | Goat |
| Tu491 | KJ495710 | Tusavirus | TN | 4424 | Human |
| GD030 | MK279318 | Bufavirus | CHN | 4189 | Porcine |
| HN019 | MK279313 | Bufavirus | CHN | 4186 | Porcine |
| /^a^ | NC_029797 | Bufavirus | INA | 4765 | Megabat |
| MAG12-57 | LC085675 | Bufavirus | INA | 4765 | Megabat |
| AH-002 | MT542983 | Bufavirus | CHN | 4219 | Canine |
| AH-003 | MT577645 | Bufavirus | CHN | 4219 | Canine |
| SY-2015 | KT716186 | Bufavirus | CHN | 4634 | Rat |
| / | NC_028650 | Bufavirus | CHN | 4634 | Rat |
| / | J02275 | Parvovirus | Not mentioned | 5149 | Mice |
| / | AF321230 | Parvovirus | US | 4904 | Rat |
| / | U44978 | Parvovirus | Not mentioned | 4948 | Porcine |
| FVP-3.us_67 | EU659111 | Parvovirus | US | 4269 | Feline |
| NA | D00765 | Parvovirus | JP | 5094 | Mink |
| CPV/Raccoon/VA/118-A/07 | JN867610 | Parvovirus | VA | 4627 | Canine |

^a^/: Not given;

^b^BF: Burkina Faso; BT: Bhutan; TUR: Turkey; DEN: Denmark; BR: Brazil; HU: Hungary; TN: Tunisia; CHN: China; INA: Indonesia; US: United States; JP: Japan; VA: Virginia.

**Supplementary Table 4**. Laboratory test datas from biochemical samples of TuV**^a^**-positive cases.

| **Items** | **GZ814**^g^ | | **GZ1068**^g^ | |
| --- | --- | --- | --- | --- |
| **Blood routine**^b^ |  |  |  |  |
| Hemoglobina (g/L) | 114 | - | 171 | - |
| Leucocitos ( × 10^9^/L) | 5.27 | - | 9.69 | - |
| Neutrophils ( × 10^9^/L) | 2.84 | - | 6.11 | - |
| Linfocitos ( × 10^9^/L) | 1.93 | - | 2.64 | - |
| Plaquetas ( × 10^9^/L) | 233 | - | 228 | - |
| CRP (mg/L) | 0.34 | - | 4.56 | - |
| **Hepatic function test**^c^ |  |  |  |  |
| AST (U/L) | 25 | - | 26 | - |
| ALT (U/L) | 17 | - | 36 | - |
| GGT (IU/L) | 16 | - | 54 | - |
| ALP (IU/L) | 91 | - | 57 | - |
| STB (μmol/L) | 9.4 | - | 17.8 | - |
| TP (g/L) | 66.7 | - | 76.9 | - |
| GLB (g/L) | 28.5 | - | 30.6 | - |
| ALB (g/L) | 38.2 | - | 46.3 | - |
| LDL-C (mmol/L) | 3.65 | - | 2.03 | - |
| HDL-C (mmol/L) | 1.67 | ↑ | 1.13 | - |
| **Renal function test**^d^ |  |  |  |  |
| Urea (mmol/L) | 4.78 | - | 8.47 | ↑ |
| CRE (μmol/L) | 45 | - | 74 | - |
| UA (μmol/L) | 217 | - | 439 | ↑ |
| **Tumor-related markers**^e^ |  |  |  |  |
| CEA (ng/ml) | 2.09 | - | 0.83 | - |
| **Fecal characteristics**^f^ |  |  |  |  |
| WBC (/HP) | 0 | - | 0 | - |
| RBC (/HP) | 0 | - | 0 | - |
| OB | Negative | - | Negative | - |
| Mucus | Negative | - | Negative | - |
| Fat droplet | Seen | ↑ | Not seen | - |
| Fungus | Not seen | - | Not seen | - |
| Parasite | Not seen | - | Not seen | - |

^a^TuV: Tusavirus;

^b^CRP: C-reaction protein;

^c^AST: aspartate aminotransferase; ALT: alanine aminotransferase; GGT: gamma glutamyl transpeptidase; ALP: alkaline phosphatase; STB: serum total bilirubin; TP: total protein; GLB: globulin; ALB: albumin; LDL-C: Low density lipoprotein cholesterol; HDL-C: High density lipoprotein cholesterol;

^d^CRE: creatinine; UA: uric acid;

^e^CEA: carcino-embryonic antigen;

^f^WBC: white blood cell; RBC: red blood cell; OB: occult blood

^g^-: normal; ↑: uptilted

**Supplementary Table 5**. The number and proportion of amino acids in VP2^a^ region of GZ814 and GZ1068.

| **Amino acid composition** | **GZ814** | | **GZ1068** | |
| --- | --- | --- | --- | --- |
|  | **number** | **proportion(%)** | **number** | **proportion(%)** |
| Cys (C) | 6 | 1.1 | 6 | 1.1 |
| Met (M) | 10 | 1.8 | 10 | 1.8 |
| Lys (K) | 15 | 2.7 | 15 | 2.7 |
| Trp (W) | 17 | 3 | 17 | 3.0 |
| His (H) | 18 | 3.2 | 18 | 3.2 |
| Phe (F) | 19 | 3.4 | 19 | 3.4 |
| Tyr (Y) | 19 | 3.4 | 19 | 3.4 |
| Glu (E) | 21 | 3.7 | 21 | 3.7 |
| Arg (R) | 23 | 4.1 | 23 | 4.1 |
| Gln (Q) | 23 | 4.1 | 23 | 4.1 |
| Asp (D) | 31 | 5.5 | 31 | 5.5 |
| Val (V) | 32 | 5.7 | 31 | 5.5 |
| Ala (A) | 32 | 5.7 | 32 | 5.7 |
| Ile (I) | 32 | 5.7 | 33 | 5.8 |
| Leu (L) | 38 | 6.7 | 38 | 6.7 |
| Ser (S) | 38 | 6.7 | 38 | 6.7 |
| Pro (P) | 42 | 7.4 | 42 | 7.4 |
| Gly (G) | 45 | 8.0 | 45 | 8.0 |
| Asn (N) | 45 | 8.0 | 45 | 8 |
| Thr (T) | 59 | 10.4 | 59 | 10.4 |
| Pyl (O) | 0 | 0 | 0 | 0 |
| Sec (U) | 0 | 0 | 0 | 0 |

^a^VP: viral protein

**Supplementary Table 6**. Phosphorylation sites of VP2^a^-TuV^b^ protein and corresponding kinases.

| **Sites** | **Score** | **Kinase** | **Sites** | **Score** | **Kinase** | **Sites** | **Score** | **Kinase** |
| --- | --- | --- | --- | --- | --- | --- | --- | --- |
| S 4 | 0.675 | unsp | S 356 | 0.994 | unsp | T 266 | 0.97 | unsp |
| S 5 | 0.524 | cdc2 | S 379 | 0.5 | DNAPK | T 280 | 0.589 | unsp |
| S 6 | 0.817 | unsp | S 388 | 0.511 | PKC | T 287 | 0.707 | PKC |
| S 8 | 0.968 | unsp | S 420 | 0.818 | unsp | T 338 | 0.507 | PKC |
| S 11 | 0.956 | unsp | S 562 | 0.512 | PKA | T 348 | 0.961 | unsp |
| S 12 | 0.97 | unsp | T 26 | 0.578 | CKII | T 352 | 0.512 | GSK3 |
| S 13 | 0.933 | unsp | T 32 | 0.547 | CKII | T 363 | 0.769 | PKC |
| S 25 | 0.989 | unsp | T 42 | 0.538 | cdc2 | T 419 | 0.599 | unsp |
| S 49 | 0.769 | PKC | T 68 | 0.619 | DNAPK | T 431 | 0.706 | PKC |
| S 60 | 0.572 | unsp | T 76 | 0.658 | PKC | T 457 | 0.881 | PKC |
| S 106 | 0.512 | p38MAPK | T 87 | 0.577 | DNAPK | T 470 | 0.758 | PKC |
| S 124 | 0.739 | unsp | T 91 | 0.585 | p38MAPK | T 494 | 0.598 | p38MAPK |
| S 177 | 0.601 | PKG | T 133 | 0.7 | PKC | T 502 | 0.522 | CKII |
| S 204 | 0.618 | RSK | T 136 | 0.557 | PKG | T 511 | 0.876 | PKC |
| S 207 | 0.995 | unsp | T 138 | 0.516 | CKI | T 563 | 0.739 | PKC |
| S 208 | 0.74 | PKC | T 140 | 0.521 | PKC | Y 63 | 0.954 | unsp |
| S 209 | 0.861 | unsp | T 171 | 0.518 | GSK3 | Y 246 | 0.925 | unsp |
| S 212 | 0.514 | cdc2 | T 179 | 0.536 | PKC | Y 316 | 0.52 | EGFR |
| S 214 | 0.947 | unsp | T 188 | 0.932 | unsp | Y 398 | 0.633 | unsp |
| S 219 | 0.612 | unsp | T 213 | 0.578 | PKC | Y 432 | 0.507 | INSR |
| S 269 | 0.564 | PKC | T 221 | 0.612 | DNAPK | Y 435 | 0.517 | SRC |
| S 281 | 0.948 | unsp | T 233 | 0.56 | DNAPK |  |  |  |
| S 355 | 0.983 | unsp | T 248 | 0.544 | PKC |  |  |  |

^a^VP: viral protein ; ^b^TuV: Tusavirus

**Supplementary Table 7**. The selection Sites of VP2^a^-TuV^b^ gene by different methods.

| **Method^c^** | **Selection sites** | | **Threshold** |
| --- | --- | --- | --- |
|  | **Positive/diversifying selection** | **Negative/purifying**  **selection** |  |
| SLAC | / | 41,63,77,85,90,92,96,115,117,119,126,130,190,196,198,203,205,215,223,230,233,234,240,245,285,314,362,378,380,386,397,399,402,403,415,416,418,419,432,438,439,448,460,470,471,473,486,492,493,512,530,560,564 | *P* < 0.1 |
| FUBAR | / | 41,63,77,85,90,92,96,115,117,119,126,130,190,196,198,203,205,215,223,230,233,234,240,245,285,314,378,380,386,397,399,402,403,415,416,418,419,432,438,439,448,460,470,471,473,486,492,493,512,530,560,564 | Posterior probability of 0.9 |
| FEL | / | 41,63,77,85,90,92,96,115,117,119,126,130,190,196,198,203,205,215,223,230,233,234,240,245,282,285,314,362,378,380,386,397,399,402,403,415,416,418,419,432,438,439,448,460,470,471,473,486,492,493,512,530,560,564 |  |
| MEME | / | 41,63,77,85,90,92,96,115,117,119,126,130,190,196,198,203,205,215,223,230,233,234,240,245,282,285,314,362,378,380,386,397,399,402,403,415,416,418,419,432,438,439,448,460,470,471,473,486,492,493,512,530,560,564 |  |

^a^VP: viral protein;

^b^TuV: Tusavirus;

^c^SLAC: Single Likelihood Ancestor Counting; FUBAR: Fast Unconstrained Bayesian AppRoximation; FEL: Fixed Effects Likelihood; MEME: Mixed Effects Model of Evolution.

**Supplementary Figure 1.** Agarose gel electrophores of PCR products of TuV positive samples screening. The PCR products were subjected to 1.0% agarose gel

electrophoresis, and visualized under a UV-transilluminator with the expected PCR product sizeof 247 bp. M: DL1000 marker (TaKaRa Biotechnology, Kusatsu, Japan) , bands representing 100 bp , 200 bp , 300 bp , 400 bp , 600 bp , 700 bp and 1000 bp length from bottom to top, respectively; a: the PCR product of GZ814; b: the PCR product of GZ1068.


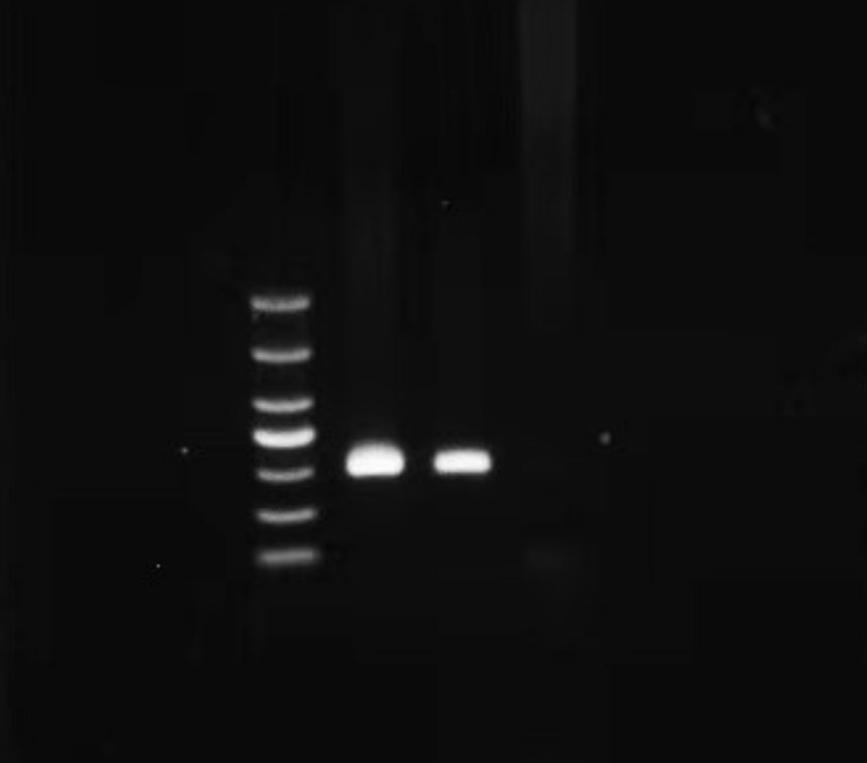


**a**

**b**

**M**

**Supplementary Figure 2.** Multi sequence comparison of VP2-TuV protein. The VP2-TuV amino acids sequences in human, sheep and goat were compared. The GenBank accession numbers of the reference sequences were provided on the left. The numbers at the bottom of the sequences represented the position of amino acid. The “*” located at the top indicated the amino acid in the position were the same. VP, viral protein; TuV, tusavirus.


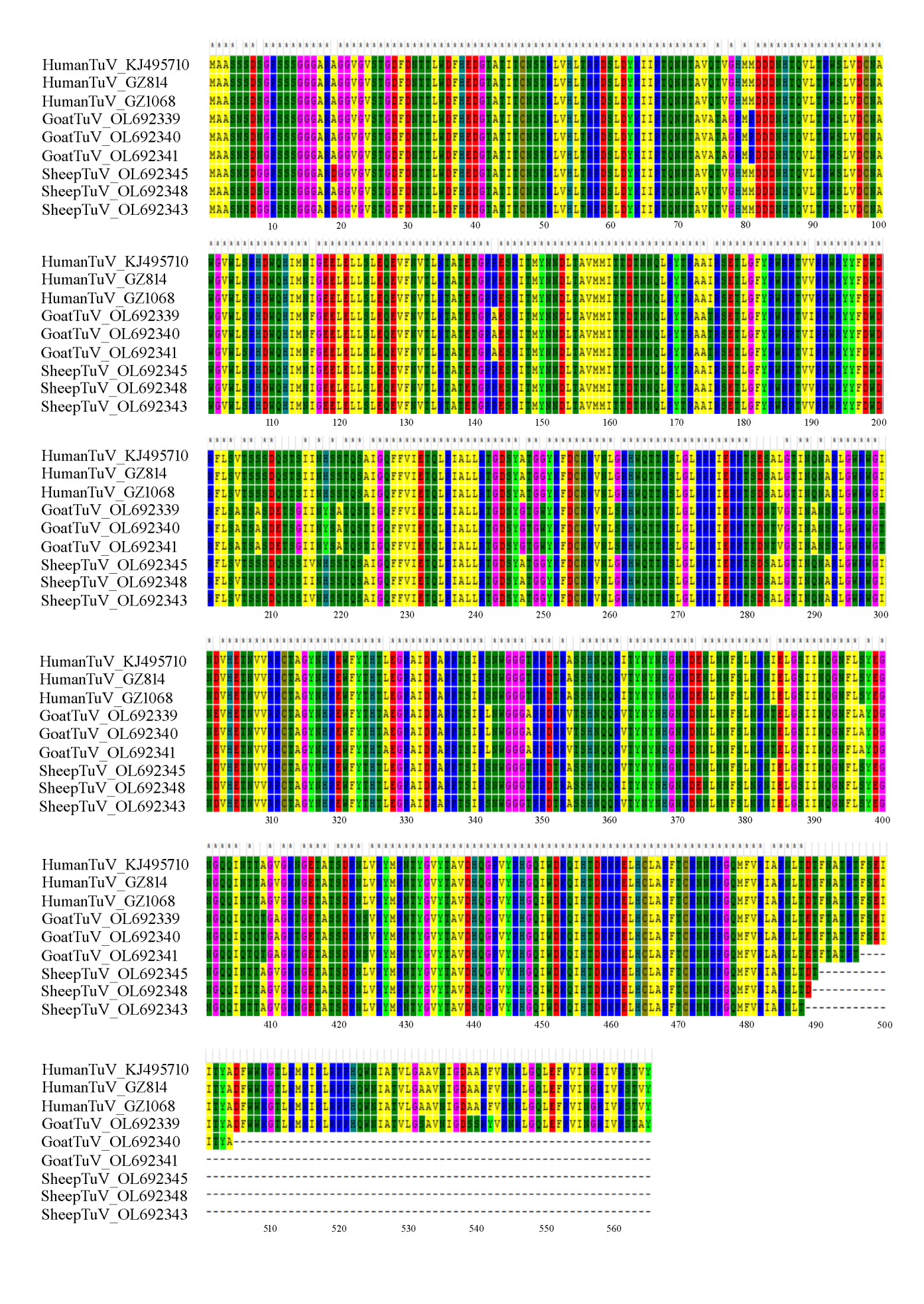


**Supplementary Figure 3.** Hydrophilcity/hydrophobicity analysis of VP2-TuV protein. The X-axis represented the position of the amino acids of the VP2-TuV protein; the Y-axis represented the score of hydrophilcity/hydrophobicity analysis. If the score > 0, indicated the amino acid in this region was hydrophobic. Conversely, if the score < 0, meant the amino acid was hydrophilic. The absolute value of the score had a positive association with the strength of hydrophilicity/hydrophobicity. VP, viral protein; TuV, tusavirus.


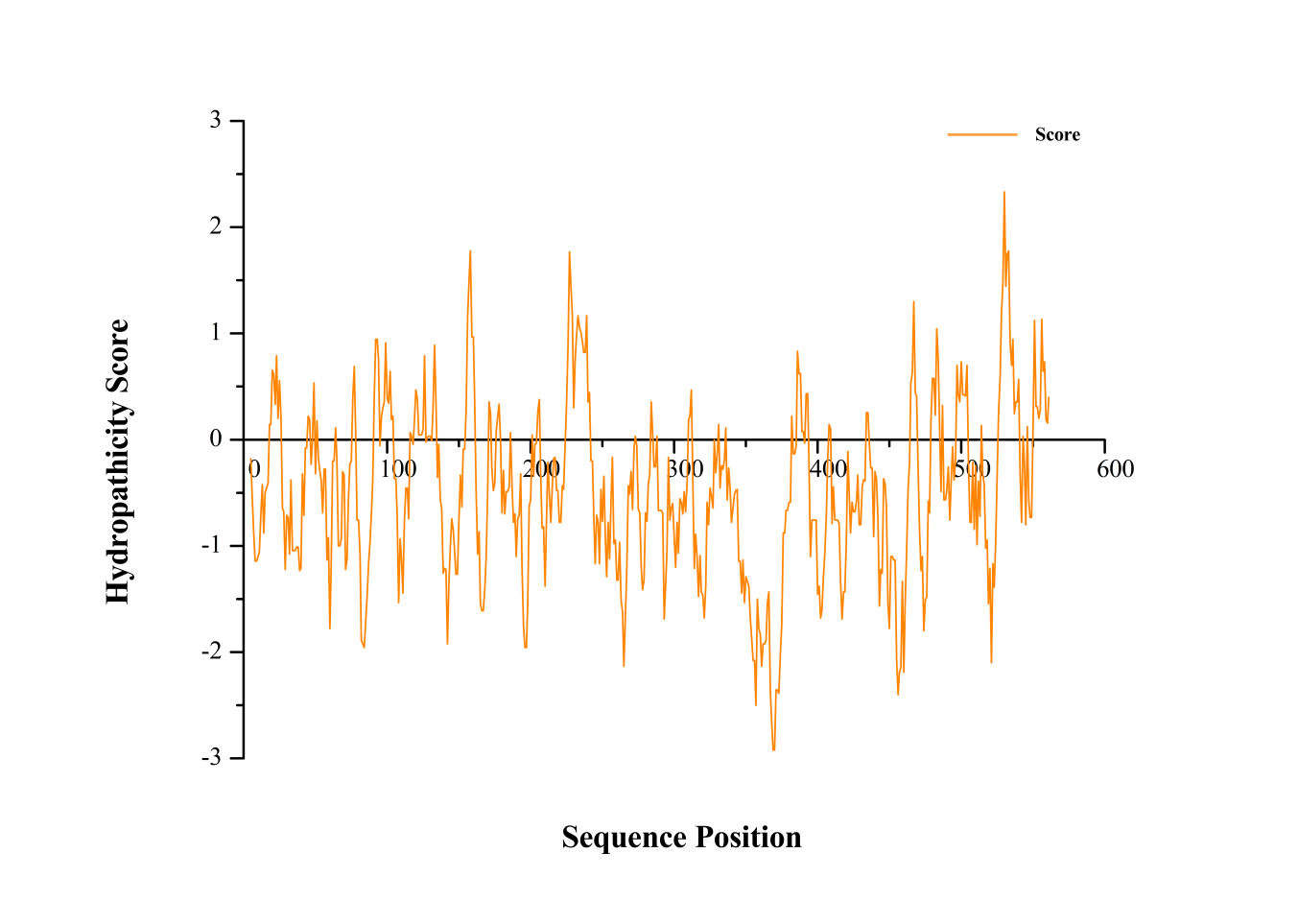


**Supplementary Figure 4.** Phosphorylation sites prediction of VP2-TuV protein. The X-axis represented the position of the amino acids of the VP2-TuV protein; the Y-axis denotesd the score of phosphorylation sites analysis. The orange horizontal line indicated the threshold, above which the result was predicted to be the phosphorylation site; different colored vertical lines represented the amino acid phosphorylation prediction scores at that site. VP, viral protein; TuV, tusavirus.


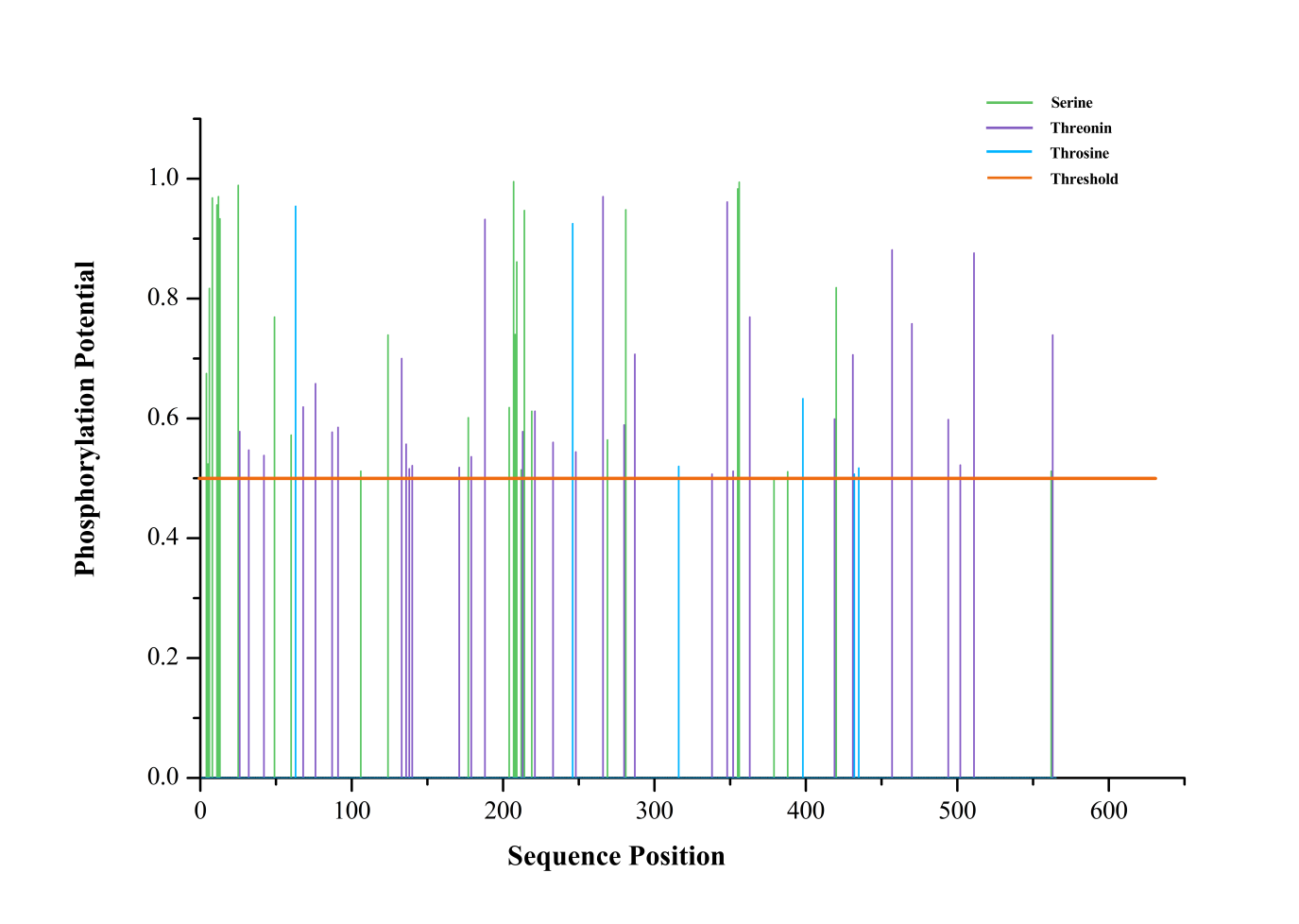


**Supplementary Figure 5.** N-glycosylation sites prediction of VP2-TuV protein. The X-axis represented the position of the amino acids of the VP2-TuV protein; the Y-axis represented the score of glycosylation sites analysis. The pink line indicated the threshold, above which the result was predicted to be the glycosylation site; the blue vertical lines representd the amino acid glycosylation prediction scores at that site. VP, viral protein; TuV, tusavirus.


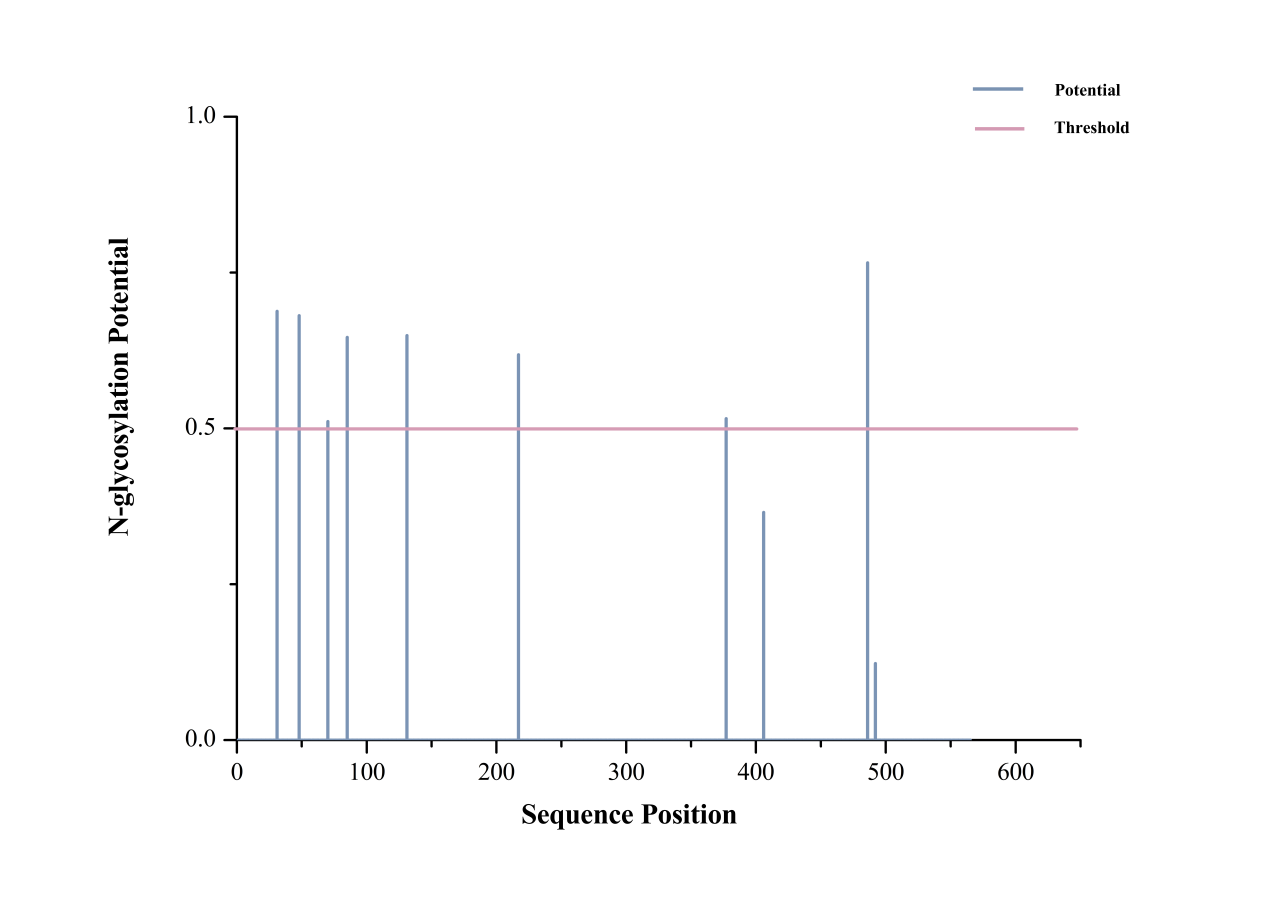


**Supplementary Figure 6.** Ramachadran plot analysis of tertiary structure prediction of human VP2-TuV protein. The X-axis showed the rotation Angle of the C-N bond to the left of the α carbon in one peptide unit and Y-axis showed the rotation Angle of the C-C bond to the right of the α carbon. The region ranged from white to red, and the darker the color, the more reasonable the dihedral Angle. Black [triangle](javascript:;)s represented Glycine; Black squares represented other amino acids except glycine. The results showed 90.7% residues were located in most favoured regions and 8.4% residues were located in additional allowed regions. VP, viral protein; TuV, tusavirus.


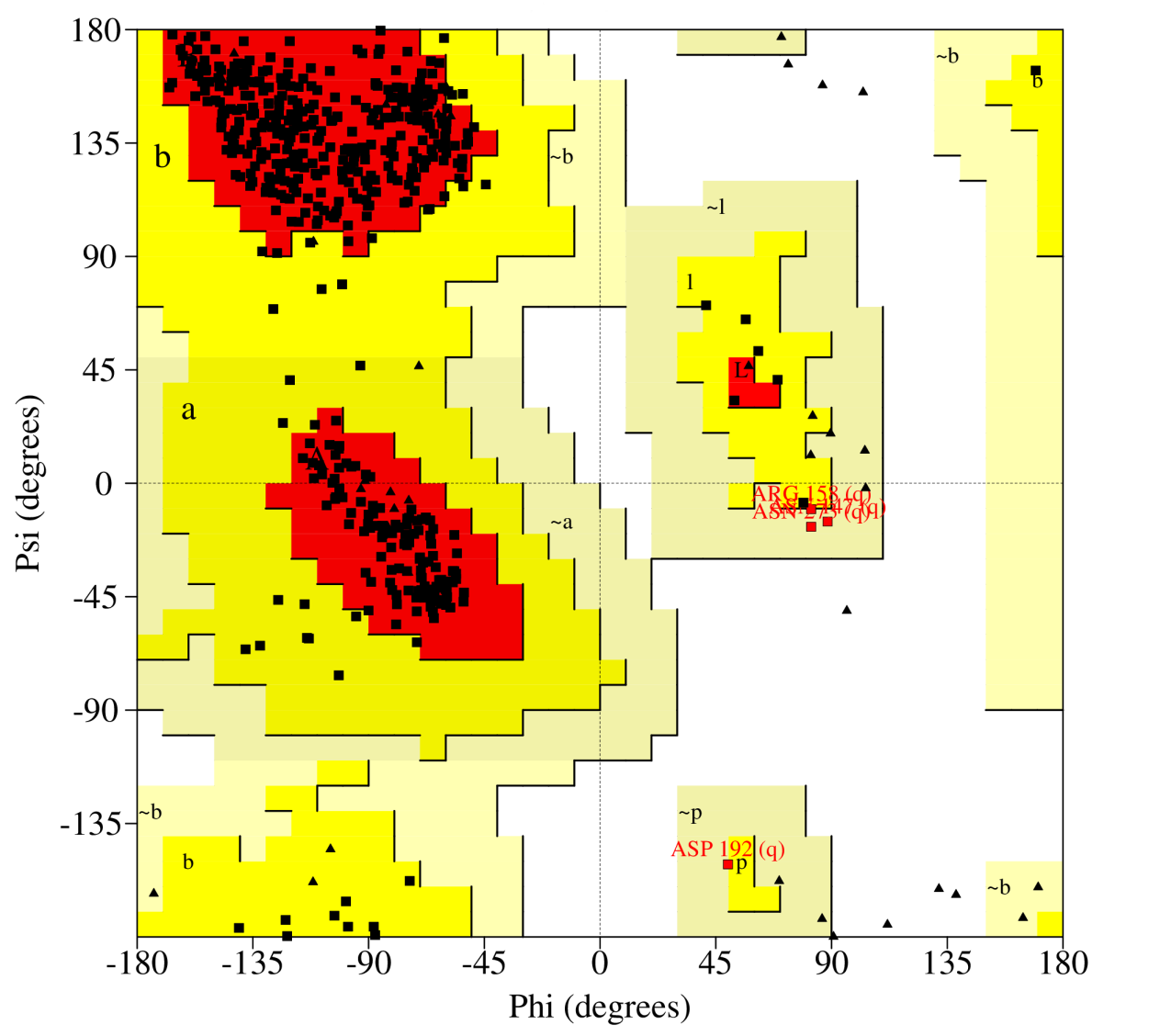

Supplement: Supplementary file 1 [file Data_Sheet_1.docx]
